# Reconstitution of Monocyte Subsets and PD-L1 Expression but Not T Cell PD-1 Expression in Obstructive Sleep Apnea Patients upon PAP Therapy

**DOI:** 10.3390/ijms222111375

**Published:** 2021-10-21

**Authors:** Christina Polasky, Armin Steffen, Kristin Loyal, Christian Lange, Karl-Ludwig Bruchhage, Ralph Pries

**Affiliations:** Department of Otorhinolaryngology, University Hospital of Schleswig-Holstein, 23538 Lübeck, Germany; christina.polasky@uksh.de (C.P.); Armin.Steffen@uksh.de (A.S.); Kristin.Loyal@uksh.de (K.L.); Christian.Lange@uksh.de (C.L.); Karl-Ludwig.Bruchhage@uksh.de (K.-L.B.)

**Keywords:** monocyte subsets, PD-1/PD-L1, obstructive sleep apnea, PAP therapy

## Abstract

Obstructive sleep apnea (OSA) is characterized by nocturnal breathing intermissions resulting in oxidative stress and eventually, a low-grade systemic inflammation. The study aimed to investigate the impact of positive airway pressure (PAP) therapy on the inflammatory milieu as measured by monocyte and T cell phenotypic alterations. Participants were assessed for their OSA severity before PAP therapy and about six months later, including patient-reported outcome and therapy usage by telemetry readout. The distributions of the CD14/CD16-characterized monocyte subsets as well as the CD4/CD8-characterized effector T cell subsets with regard to their PD-1 and PD-L1 expression were analyzed by flow cytometry from blood samples. Data of 25 patients revealed a significant reconstitution of the monocyte subset distribution and a decrease in PD-L1 expression on pan-monocytes and CD8^+^ T cells without an association to initial AHI and overweight. The PD-1 expression was still increased on T cell subsets, especially on CD4^+^ TH17/22 cells. We conclude that PAP therapy might have a rapid effect on the monocyte phenotype and overall PD-L1 expression levels. However, T cell immune alterations especially on TH17/22 cells persist longer, indicating an ongoing disturbance of the adaptive immune system.

## 1. Introduction

Obstructive sleep apnea (OSA) represents a substantial and frequent disease of recurrent breathing intermissions, resulting in a partial (hypopnea) or complete cessation (apnea) of the airflow in the upper airways followed by reoxygenation [1]. The disease leads to intermitted hypoxia and nocturnal oxidative stress, which trigger a low-grade systemic inflammation. This condition promotes an activation of different immune cells such as lymphocytes and monocytes that secrete high amounts of inflammatory cytokines and adhesion molecules [2,3]. The current standard treatment for OSA is a noninvasive respiratory support tool that provides positive airway pressure (PAP) [4]. A nighttime usage of 4 h per night has been considered the minimum to improve daytime performance measured by the ESS (Epworth Sleepiness Scale) of patients. It has been shown to improve daytime sleepiness, quality of life, and depressive symptoms, as well as reduce risk for cardiovascular disease and mortality [5,6,7,8], whereas the impact on the last mentioned is highly debated [9].

In the past decade, it has become increasingly obvious that OSA-induced oxidative stress stands in a clear context with changes in B, T and NK cell composition and activation of certain lymphocyte subsets [10,11,12,13].

We have recently shown that OSA patients reveal decreased levels of CD14^++^CD16^−^ classical monocytes accompanied by an increase in both CD16^+^ monocyte subsets and an imbalanced PD-1/PD-L1 cross-talk with CD4/CD8 T cells. [14].

Monocytes have been subdivided based on their CD14 and CD16 expression levels [15,16,17]. CD14^++^CD16^−^ “classical” monocytes are considered as “naïve-like” monocytes that are released from the bone marrow into the periphery. The CD14^+^CD16^+^ “intermediate” and CD14^dim+^CD16^+^ “non-classical” monocytes are assumed to be more differentiated, activated, pro-inflammatory monocyte subsets that exert specialized functions such as viral defense, professional antigen presentation and patrolling behavior [18,19]. Under physiological conditions, they each comprise an amount of 5–10% of peripheral blood monocytes. An increase in these pro-inflammatory subsets has been proven in various acute and chronic inflammatory diseases [20,21,22].

The aim of this study was to understand the impact of PAP therapy in patients with OSA on differentiation patterns of circulating monocytes with regard to the three above described subsets. Furthermore, a detailed analysis of the CD4/CD8 T cell subset composition, especially with respect to the immune checkpoint molecules PD-1 (programmed death-1) and PD-L1 (programmed death ligand-1), was addressed, because an upregulated PD-1/PD-L1 crosstalk is known to suppress T cell activation. Immunological data were compared to clinical parameters and quality of life of the patients. The study aimed to broaden our understanding on the immunological impact of oxidative stress and OSA treatment of it on a cellular level.

## 2. Results

### 2.1. Patients Characteristics and Response to PAP Therapy

The cohort analysis of 25 patients displayed an almost gender-balanced, middle-aged patient group with predominantly mild OSA. About 48% of the patients suffered from a mild OSA (AHI 5–14/h), 44% from a moderate (15–30/h), and 8% presented a severe OSA (≥30/h). Initial ESS assessment showed a mean of 10 points; below 10 is regarded as non-suspicious for German normative values [23]. Mean ESS after therapy was 8 (±3.4). According to WHO guidelines, 2 patients displayed a normal weight, whereas 23 patients were overweight, of which 14 had overweight (BMI 26–30), 5 had adiposity I°, (BMI 31–35) and 4 had adiposity II° (BMI 36–40).

The average usage time of PAP therapy was 4.8 h (±2.3 h) per night over the last 30 days and 5.1 h (±2 h) per night over the last 90 days of therapy. Six patients used the therapy for less than 4 h per night over the last 30 days and four of them used it less than 4 h over the last 90 days. Parameters are summarized in Table 1.

### 2.2. Monocyte Subset Distribution in Response to PAP Therapy

The gating strategy of CD14- and CD16-defined monocyte subsets was performed as published before. CD14^++^CD16^−^ (classical), CD14^++^CD16^+^ (intermediate) and CD14^dim+^CD16^+^ (non-classical) monocyte subsets were distinguished. PD-L1 expression on pan-monocytes was determined from isolated PBMC.

Analyzed OSA patients could be subdivided based on the initial severity of monocyte alterations before respiratory support. Most patients (*n* = 18) revealed a moderate decrease in classical monocytes accompanied by an increase in intermediate monocytes. Seven patients showed a severe drop in classical monocytes with a strong increase in intermediate and non-classical monocytes in comparison to healthy donors [14]. The monocyte subset distribution was not correlated to the BMI or AHI of the patients.

**Figure 1:**
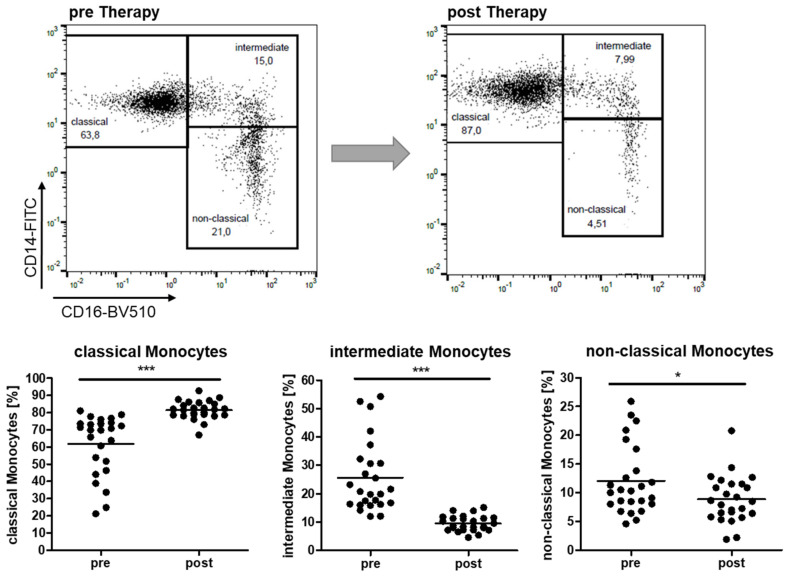
Flow cytometric analysis of CD14- and CD16-characterized monocyte subsets. Shown are analyses of one representative patient before and after 6 months PAP therapy. Dot plots show percentages of classical, intermediate and non-classical monocytes before (pre) and after (post) therapy. *n* = 25. *: *p* < 0.05; and ***: *p* < 0.001.

Our investigations identified a highly significant reconstitution of the observed alterations of the abundance of all three monocyte subsets in all OSA patients after PAP therapy for at least 6 months. Flow cytometry data revealed a significant increase in classical monocytes accompanied by a drop in intermediate and non-classical subsets in all analyzed patients (Figure 1). Most of the patients even revealed monocyte distributions comparable to healthy donors in response to PAP [14]. 

**Figure 2:**
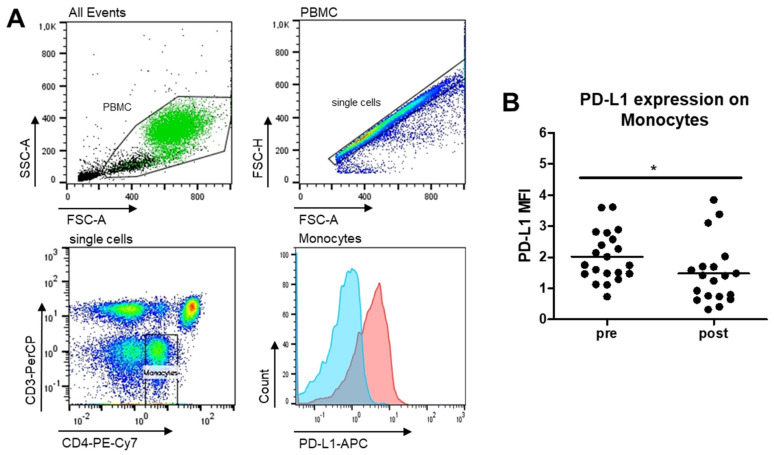
Flow cytometric analysis of monocytes in PBMC from OSA patients before and after PAP treatment. (**A**): Gating scheme of monocytes (green) within the PBMC fraction. Gating was performed by the help of low CD4 expression of monocytes, lack of CD3 and their FSC/SSC characteristics. Histogram shows expression profile of one representative patient before (red peak) and after (blue peak) therapy. (**B**): PD-L1 expression intensity (MFI) on total monocytes measured as CD4^low+^CD3^−^ cells in isolated PBMCs. *n* = 25. *: *p* < 0.05.

Additionally, PD-L1 expression on whole monocytes was significantly decreased after PAP therapy when compared to prior therapy (Figure 2).

### 2.3. Distribution of T Cell Subsets in Response to PAP Therapy

**Figure 3:**
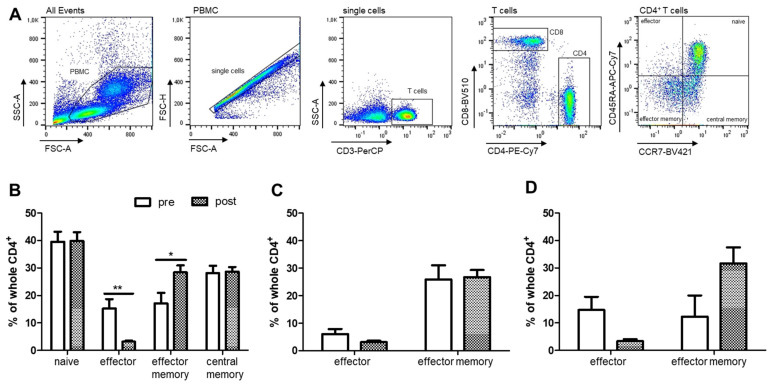
Flow cytometric analysis of CD4^+^ T cell subsets in PBMC from OSA patients (*n* = 22) before and after PAP treatment. (**A**): Gating scheme of CD4^+^ T cell subsets from one representative patient. (**B**): Percentages of naïve, effector, effector memory and central memory CD4^+^ T cells. (**C**): Percentage of effector and effector memory CD4^+^ T cells in patients with initially moderate monocyte alterations. (**D**): Percentage of effector and effector memory CD4^+^ T cells in patients with initially severe monocyte alterations. *: *p* < 0.05; **; *p* < 0.01.

Subsequent investigations of T cell subsets in PAP-treated OSA patients were carried out by FACS analysis of isolated PBMC. The T cell differentiation from naïve to effector, effector memory and central memory cells was analyzed for CD4^+^ and CD8^+^ T cells by specific markers (Figure 3A). The percentages of each subset as well as the PD-1 and PD-L1 expression levels were analyzed. 

Data revealed a significant decrease in the percentage of CD4^+^ effector T cells accompanied by an increase in effector memory T cells after PAP therapy (Figure 3B). Of note, T cell reconstitution was most pronounced in patients with severe monocyte alteration prior therapy, although changes were not significant (Figure 3C vs. Figure 3D). 

**Figure 4:**
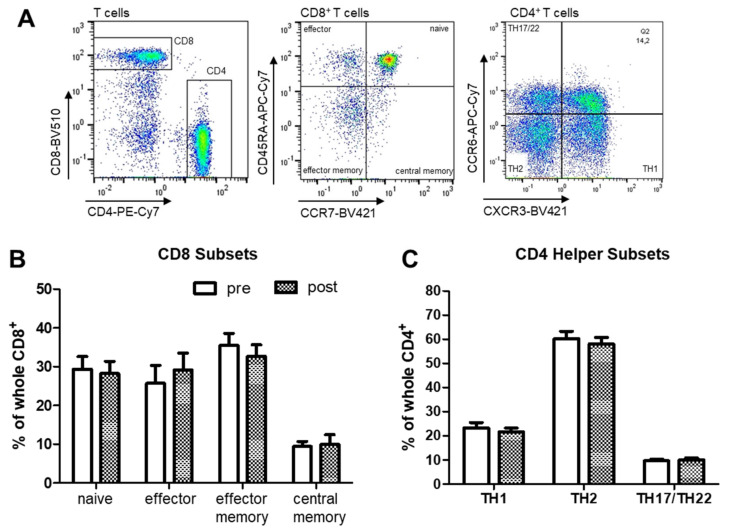
Flow cytometric analysis of CD8^+^ and CD4^+^ T helper cell subsets in PBMC from OSA patients (*n* = 22) before and after PAP treatment. (**A**): Part of the gating scheme of CD8^+^ and CD4^+^ T cell subsets. (**B**): Percentages of naïve, effector, effector memory and central memory T cells within CD8^+^ T cells. (**C**): Percentages of CD4^+^ T helper cell subsets TH1, TH2 or TH17/22. There was no statistical significance (*p* > 0.05).

CD8^+^ T cell subtypes were not significantly altered in response to respiratory support (Figure 4B) and analysis of patient groups regarding an initial moderate or severe monocyte alteration revealed no further results. The analyses of the CD4^+^ T helper type subsets (TH1, TH2, TH17/TH22) showed no differences in the percentages of each subtype (Figure 4C).

**Figure 5:**
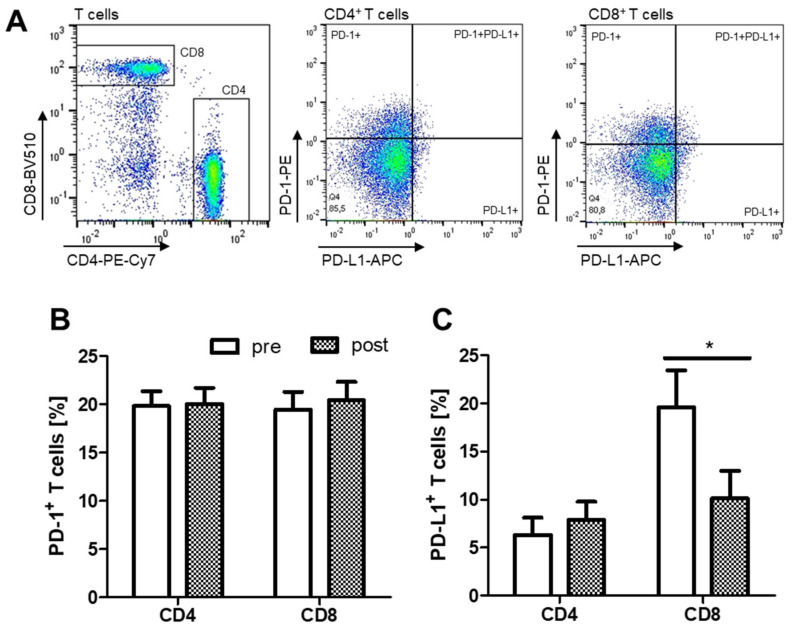
Flow cytometric analysis of CD4^+^ and CD8^+^ T cell subsets in PBMC from OSA patients (*n* = 22) before and after PAP treatment. (**A**): Gating scheme of PD-1^+^ and PD-L1^+^ cells within the CD4^+^ and CD8^+^ T cell subset. (**B**): Percentages of PD-1^+^ cells within the whole CD4^+^ or CD8^+^ T cell subset. (**C**): Percentages of PD-L1^+^ cells within the whole CD4^+^ or CD8^+^ T cell subset. *: *p* < 0.05.

The percentages of PD-1^+^CD4^+^ as well as PD-1^+^CD8^+^ were not changed after PAP therapy. The percentage of PD-L1^+^CD8^+^ T cells was significantly decreased after PAP therapy (Figure 5B).

**Figure 6:**
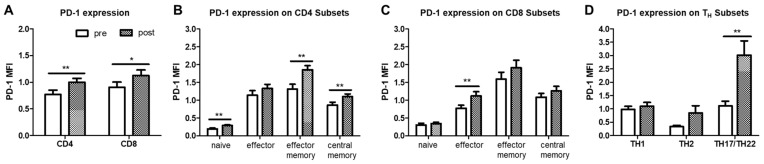
Flow cytometric analysis of CD4^+^ and CD8^+^ T cell subsets in PBMC from OSA patients (*n* = 22) before and after PAP treatment. (**A**): PD-1 expression intensity (MFI) of whole CD4^+^ or CD8^+^ T cell subsets. (**B**): PD-1 expression intensity (MFI) on naïve, effector, effector memory and central memory T cells within CD4^+^ T cells. (**C**): PD-1 expression intensity (MFI) on naïve, effector, effector memory and central memory T cells within CD8^+^ T cells. (**D**): PD-1 expression intensity (MFI) on TH1, TH2 or TH17/22 T helper cell subsets. MFI = mean fluorescence intensity. *: *p* < 0.05; **: *p* < 0.01.

The PD-1 expression intensity (MFI) was found to be significantly increased overall on CD4^+^ and CD8^+^ T cells in OSA patients after PAP therapy when compared to prior therapy (Figure 6A). Changes were most pronounced on CD4^+^ naïve, effector memory and central memory cells (Figure 6B). In CD8^+^ T cells, only effector cells showed a significant increase (Figure 6C). By screening the CD4^+^ T helper type subsets, TH17/TH22 cells were found to display the highest increase in PD-1 expression after therapy (Figure 6D). 

**Figure 7:**
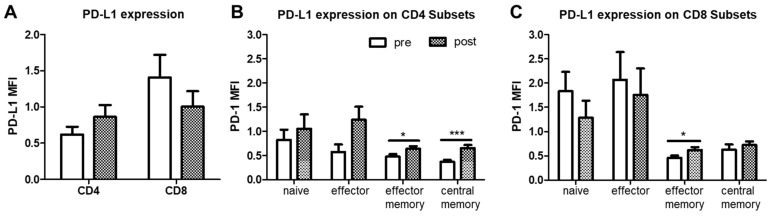
Flow cytometric analysis of CD4^+^ and CD8^+^ T cell subsets in PBMC from OSA patients (*n* = 22) before and after PAP treatment. (**A**): PD-L1 expression intensity (MFI) whole CD4^+^ or CD8^+^ T cell subsets. (**B**): PD-L1 expression intensity (MFI) on naïve, effector, effector memory and central memory T cells within CD4^+^ T cells. (**C**): PD-L1 expression intensity (MFI) on naïve, effector, effector memory and central memory T cells within CD8^+^ T cells. MFI = Mean fluorescence intensity. *: *p* < 0.05; ***: *p* < 0.001.

Analysis of the PD-L1 expression intensity on T cells revealed a significant increase on effector memory and central memory CD4^+^ T cells after PAP therapy (Figure 7B). On CD8^+^ T cells, PD-L1 expression was likewise increased on the effector T cell subset in response to PAP therapy (Figure 7C). Percentages of PD-L1^+^ T cells were not altered. 

**Figure 8:**
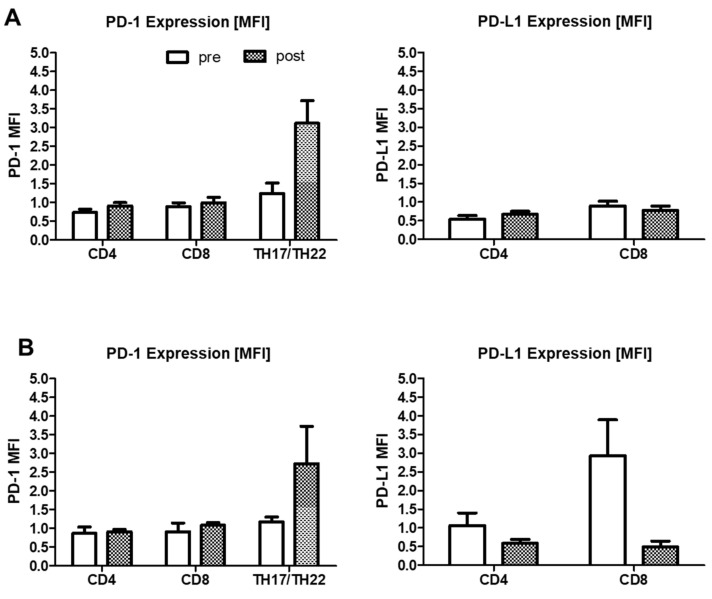
Flow cytometric analysis of CD4^+^ and CD8^+^ T cell subsets in PBMC from OSA patients before and after PAP treatment divided by initial severity of monocyte alteration. (**A**): PD-1 and PD-L1 expression intensity on TH17/22^+^ CD4^+^ T cells or total CD4^+^ and CD8^+^ T cells from patients of the moderate monocyte alteration group. (**B**): PD-1 and PD-L1 expression intensity on TH17/22^+^ CD4^+^ T cells or total CD4^+^ and CD8^+^ T cells from patients of the severe monocyte alteration group. MFI = mean fluorescence intensity. There was no statistical significance.

When analyzing the patient groups regarding the initial monocyte alteration severity, PD-1 expression was mostly increased on TH17/TH22 cells after PAP therapy in both groups independently of the initial monocyte percentages (Figure 8A vs. Figure 8B left panel). Comparison of these groups with respect to their PD-L1 expression revealed a more pronounced decrease in PD-L1 expression on CD8^+^ T cells in the initially severe group, whereas the moderate group was not altered (Figure 8A vs. Figure 8B right panel). 

## 3. Discussion

The present study was undertaken to investigate the impact of PAP therapy on percentages of monocyte subsets and T cell immune alterations, distinguishing between CD4^+^ and CD8^+^ T cells. Additionally, the expression levels of PD-1 and PD-L1 on the peripheral T cells of OSA patients were analyzed for CD3^+^CD4^+^ and the CD3^+^CD8^+^ subsets and monocytes using flow cytometry.

In a recent study, we have shown that OSA triggers a significant decrease in classical monocytes accompanied by an increase in CD16^+^ subsets [14]. Similar high percentages of intermediate monocytes were, up to now, only found in asthma patients [21]. An increase in pro-inflammatory CD16^+^ monocyte subsets has been linked to various inflammatory conditions [15]. OSA-related oxidative stress promotes the upregulation of pro-inflammatory transcription factors such as NFκB and HIF-1α [24]. The impact of PAP therapy in OSA patients has been so far solely evaluated on cytokine levels and mediators linked to atherosclerosis, because this is the most common secondary disease of OSA [25]. Several studies already proved significantly increased TNF-α, CRP, IL-6, IL-8, VCAM, ICAM, and E-Selectin levels in OSA patients, which decreased after at least 3 months of PAP therapy [26,27,28]. Data from the large cohort MOSAIC study did, on the other hand, not show any significant changes in inflammatory parameters after 6 months of CPAP therapy [29].

The present study is the first to address OSA-induced immunological changes with respect to the influence of PAP therapy at a cellular level. We found a normalization of the monocyte subset distribution after PAP therapy in all patients (Figure 1). We assume that the oxygenation-mediated decrease in the previously mentioned cytokines is responsible for the recovery of monocyte subsets or at least the abandoned differentiation towards inflammatory cells. We additionally found a significant correlation of the BMI of patients with the percentage of classical monocytes in our previous study and, therefore, concluded that the obesity-associated low-grade systemic inflammation acts as a cofactor to oxidative stress on monocyte alterations. Since none of the patients lost weight during PAP therapy, but monocyte alteration was reversed to normal levels, the impact of overweight might not be as relevant as assumed. These observations go in line with a study from Ng and colleagues, which found significantly decreased adiponectin and irisin levels in obese OSA patients that were treated with PAP for 3 months [30]. Better oxygenation might have a positive effect on adiposity-related cytokine levels, even without weight loss.

Additionally, a clear connection of oxidative stress and increased PD-L1 expression has been proven in several studies [10,11]. OSA patients from our study revealed likewise an increased PD-L1 expression on monocytes and CD8^+^ T cells at initial diagnosis, which was reversed to levels of healthy donors after PAP therapy (Figure 2; Figure 5C). Of note, seven patients with initially more severe monocyte subset alterations showed the most pronounced improvement by tendency (Figure 8B). A higher expression of PD-L1 on CD8^+^ T cells was also initially linked to monocytic PD-L1 expression and the constitution of monocyte subsets [13]. These findings are most probably related to the better oxygenation during night and abolished oxidative stress as seen by the improved AHI. Surprisingly, levels of PD-1 expression were even more increased on CD4^+^ and CD8^+^ T cells after PAP therapy. TH17/TH22 cells revealed the highest PD-1 expression (Figure 6D). Whether these findings are connected to ongoing stronger T cell activation or exhaustion need to be examined in further studies. In general, our data indicate that the phenotype of innate immune cells is rapidly influenced by PAP therapy, whereas effects on cells of the adaptive immune system are longer lasting. Despite percentages of naïve and effector CD4^+^ T cells being recovered to healthy levels, the amount of central memory cells is still decreased in comparison to healthy donors [14], which may point towards an ongoing higher susceptibility to recurrent infections. Again, patients with more severe monocyte subset alterations showed the most pronounced shifts in T cell subsets (Figure 3D). As these alterations were strongly connected prior therapy, patients with the strongest normalization of monocyte subsets also revealed the most pronounced changes in T cells. Nevertheless, the molecular mechanisms regarding OSA-related T cell alterations and their interplay with monocytes have to be addressed in future studies.

Interestingly, none of the measured immunological parameters showed a correlation to the AHI before therapy, the BMI, or the mean usage of the PAP mask. Additionally, patients with less than 4 h PAP usage per night showed a reconstitution of CD14/CD16-characterized monocyte subsets and partial improvements in T cell parameters. The nocturnal usage time might not be as important for changes at the immunological level. Alternatively, monocytes as innate immune cells with a high daily fluctuation might not be a sufficient marker population to estimate immunological disturbances and their changes upon therapy. We conclude that monocytes together with more adaptive, long-living immune cells give more sufficient information about immune alterations. PAP therapy in general showed a quite limited tolerance and effectiveness amongst examined patients. The usage time was rather low for most patients and the improvement in daytime sleepiness was likewise only minimal. This might be connected to the still recurrent awakenings during night due to the uncomfortable mask. For a lot of patients, a therapy with an electronic device stimulating the tongue seems to be more convenient and effective.

In conclusion, the present study shows that the respiratory support of OSA patients efficiently counteracts the oxidative stress-related imbalance in circulating monocytes and PD-L1 levels, whereas the impact on T cells remains elusive. We conducted a first pilot study on OSA-induced immunological changes at the cellular level and were able to prove some interesting alterations. The small sample size has to be mindfully considered and points out the need for more comprehensive studies on a larger patient cohort in the future.

## 4. Materials and Methods

### 4.1. Characteristics of Examined OSA Patients

We enrolled all OSA patients with an indication for PAP therapy, which was based on the OSA severity by the apnea hypopnea index (AHI), their non-restorative sleep expressed by daytime sleepiness, and/or comorbidities [31]. Patients with dominant central sleep apnea or previous PAP therapy within the last six months before initiation were excluded. For the pilot character of this study, there have been no restrictions regarding AHI and overweight, measured as body mass index (BMI). Initial OSA diagnosis was based on home sleep testing devices, and in isolated patients, with polysomnography. About six months after PAP initiation, patients were invited for an office-based follow-up including the readout of their PAP devices and the ESS questionnaire. The study was approved by the local ethics committee (16-278).

Twenty-five patients were enrolled in the study and signed an informed written consent. The patient cohort had a medium age of 51 (±13) years and included 15 men and 10 women. In total, 52% also had arterial hypertension, 20% suffered additionally from cardiomyopathy, arrhythmia or myocardial infarction and 4% displayed type 2 diabetes. Average use of the PAP mask per night over the last 90 days was evaluated as well. Clinical parameters of the patients are summarized in Table 1.

### 4.2. FACS Analysis of Monocytes and T Cells

Blood was drawn by venipuncture into a sodium citrate containing S-Monovette (Sarstedt; Nümbrecht, Germany). Flow cytometric analyses were performed as described previously [14]. In short, monocyte subsets were analyzed in whole blood by help of the markers CD45, HLA-DR, CD14 and CD16. CD3^+^CD4^+^ and CD3^+^CD8^+^ T cell subsets were analyzed in isolated PBMC from the remaining blood. Furthermore, effector cell populations and T helper cell subsets were characterized by help of their CD45RA and CCR7 expression or CCR6 and CXCR3, respectively. PD-1 as well as PD-L1 were used as activation markers on T cells. Monocytes were gated as CD4^dim+^CD3^−^ cells within the PBMC fraction and PD-L1 expression was analyzed as well. Flow cytometry was performed using a MACSQuant 10 flow cytometer (Miltenyi Biotec, Bergisch-Gladbach, Germany) and data were analyzed using the FlowJo^TM^ software version 10.0 (FlowJo, LLC, Ashland, Wilmington, DE, USA). Gating of monocyte and T cell subsets was exactly performed as described before [14].

### 4.3. Statistical Analysis

Statistical analyses were performed with GraphPad Prism Version 7.0f (GraphPad Software, Inc., San Diego, CA, USA). The mean and standard error (SEM) are presented. Statistical analyses were performed using paired Student’s *t*-tests for pairwise comparison of data before and after PAP treatment; *p* < 0.05 (*), *p* < 0.01 (**), and *p* < 0.001 (***). Additional statistical details are given in the respective figure legends, when appropriate.

## Figures and Tables

**Table 1:** Clinical parameters of the 25 patients as median AHI, ESS and BMI. For the post therapy AHI, mean values measured over the last 30 days of therapy were used. Differences in AHI and ESS compared to prior therapy were expressed as ΔAHI and ΔESS. Values are presented as mean ± SD. AHI per hour less than or equal 5.0 is defined as normal. According to WHO standards, overweight is defined as a BMI greater than or equal to 25; ESS less than or equal to 9.0 is defined as normal.

	Pre Therapy (*n* = 25)	Post Therapy (*n* = 25)
AHI (per h)	19.4 (±20.0)	1.1 (±1.1)
Δ AHI 30 days		17.6 (±21.6)
ESS	10 (±3.7)	8 (±3.4)
Δ ESS		1.2 (±3.8)
BMI (kg/m^2^)	30.2 (±4.9)	30.1 (±4.7)

## Data Availability

The data presented in this study are available on request from the corresponding author. The data are not publicly available due to the privacy policy of the University Hospital Schleswig Holstein.
